# The prevalence of diabetes-related complications and multimorbidity in the population with type 2 diabetes mellitus in the Basque Country

**DOI:** 10.1186/1471-2458-14-1059

**Published:** 2014-10-10

**Authors:** Edurne Alonso-Morán, Juan F Orueta, Jose Ignacio Fraile Esteban, José M Arteagoitia Axpe, M Luz Marqués González, Nuria Toro Polanco, Patxi Ezkurra Loiola, Sonia Gaztambide, Roberto Nuño-Solinis

**Affiliations:** O + berri, Basque Institute for Healthcare Innovation, Torre del BEC (Bilbao Exhibition Centre), Ronda de Azkue 1, 48902 Barakaldo, Spain; Osakidetza, Basque Health Service, Astrabudua Health Centre, Mezo 35, 48950 Erandio, Spain; Osakidetza, Basque Health Service, San Miguel Health Centre, Garbileku s/n, 48970 Basauri, Spain; Department of Health, Donostia-San Sebastián 1, 01010 Vitoria-Gasteiz, Spain; Osakidetza, Basque Health Service, Uribe Country Manager, Alango 30, 48992 Getxo, Spain; Osakidetza, Basque Health Service, Zumaia Health Centre, Basadi 15, 20750 Zumaia, Spain; Endocrinology and Nutrition Department, Osakidetza, Basque Health Service, Cruces University Hospital, Plaza de Cruces s/n, 48903 Barakaldo, Spain; The University of the Basque Country, Leioa, Biscay, Spain; Spanish Biomedical Research Centre in Diabetes and Associated Metabolic Disorders, CIBERDEM, Madrid, Spain

**Keywords:** Diabetes-related complications, Diabetes-unrelated pathologies, Multimorbidity, Type 2 diabetes

## Abstract

**Background:**

Type 2 diabetes mellitus is associated with a diverse range of pathologies. The aim of the study was to determine the incidence of diabetes-related complications, the prevalence of coexistent chronic conditions and to report multimorbidity in people with type 2 diabetes living in the Basque Country.

**Methods:**

Administrative databases, in four cross sections (annually from 2007 to 2011) were consulted to analyse 149,015 individual records from patients aged ≥35 years with type 2 diabetes mellitus. The data observed were: age, sex, diabetes-related complications (annual rates of acute myocardial infarction, major amputations and avoidable hospitalisations), diabetes-related pathologies (prevalence of ischaemic heart disease, renal failure, stroke, heart failure, peripheral neuropathy, foot ulcers and diabetic retinopathy) and other unrelated pathologies (44 diseases).

**Results:**

The annual incidence for each condition progressively decreased during the four-year period: acute myocardial infarction (0.47 to 0.40%), major amputations (0.10 to 0.08%), and avoidable hospitalisations (5.85 to 5.5%). The prevalence for diabetes-related chronic pathologies was: ischaemic heart disease (11.5%), renal failure (8.4%), stroke (7.0%), heart failure (4.3%), peripheral neuropathy (1.3%), foot ulcers (2.0%) and diabetic retinopathy (7.2%). The prevalence of multimorbidity was 90.4%. The highest prevalence for other chronic conditions was 73.7% for hypertension, 13.8% for dyspepsia and 12.7% for anxiety.

**Conclusions:**

In the type 2 diabetes mellitus population living in the Basque Country, incidence rates of diabetes complications are not as high as in other places. However, they present a high prevalence of diabetes related and unrelated diseases. Multimorbidity is very common in this group, and is a factor to be taken into account to ensure correct clinical management.

## Background

The prevalence of type 2 diabetes is increasing worldwide [1], probably due to a longer life expectancy of the general population, a sedentary lifestyle and, above all, to increasing obesity. Prevalence in Spain has been estimated at 13.8% (43% unknown diabetes) [2].

Diabetes is a chronic disease that leads to high morbidity and mortality resulting from the complications that develop during its clinical course. Patients with diabetes are twice as likely to develop cardiovascular disease compared to the general population of the same age and sex, and this risk remains the same after adjustments for other traditional cardiovascular risk factors (CVRFs) [3]. Moreover, it is the leading cause of death in 80% of patients with diabetes in comparison to 30% in the general population [4]. This excess mortality is higher in women than in men [3, 5, 6], and life expectancy is shortened by 7-10 years [7].

Incidences of chronic diabetes-related complications have been related to poor metabolic control, disease duration and the presence of associated CVRFs [8–10], although these factors account for only a part of this increased cardiovascular risk [6]. Early and aggressive management of the disease is required to reduce cardiovascular events and improve prognosis [11] which, in the Spanish context, is supported by means of the strategic plan developed by the Spanish National Health System (SNS) [12].

An association has been found between the increase in comorbidities and a greater use of health services [13–15]. Many patients with type 2 diabetes also have other chronic diseases interfering in the management of the disease, patient education and affecting health outcomes. Previous studies have examined coexisting chronic pathologies in patients with type 2 diabetes mellitus [16, 17].

Therefore, this study aims to determine the prevalence and incidence of diabetes-related complications, as well as to provide a description of the multimorbidity of this group in the Basque Country.

## Methods

The study protocol was approved by the Clinical Research Ethics Committee of Euskadi (PI2014074), Spain. Informed consent was not obtained because patient health records were made anonymous and deidentified prior to analysis.

A descriptive study was performed which included all patients with type 2 diabetes mellitus treated at Osakidetza - the Basque Health Service. The Basque Autonomous Community (BAC) has a public and universal health system, where the Department of Primary Care acts as a gatekeeper to access to other departmental services.

For this study, the demographic and clinical variables information was obtained from the PREST database. In such database, diagnoses are coded according to the International Classification of Diseases (ICD-9-CM) [18], and the coding system used for drugs is the *Anatomical, Therapeutic, Chemical (ATC) classification system* [19]. PREST is the database of the Stratification Programme which commenced in 2010 with the aim of classifying all Basque citizens using the Johns Hopkins Adjusted Clinical Groups (ACGs) case-mix system. This case mix enables health problems to be identified from diagnoses and prescriptions and patients to be categorised according to their healthcare needs. PREST therefore combines information from different sources (primary, specialty, emergency and inpatient care) to obtain clinical variables, prescriptions and procedures. A more detailed description is found in previous publications [20].

The identification of all patients with type 2 diabetes mellitus was based on selecting everybody who, according to the PREST database, had been diagnosed with type 2 diabetes mellitus or unspecified diabetes mellitus (including its complications) at any point in their lives, or who had been prescribed antidiabetic medication, regardless of whether or not they had visited healthcare services during the observation period (September 2007 - August 2011). Excluded from this group were patients who had been diagnosed with type 1 diabetes mellitus during any contact with the services, or those whose diagnosis always corresponded to unspecified diabetes mellitus, but the only medication received was insulin. Although type 2 diabetes can occur at any age [21], it is more common after age 40; patients aged under 35 were excluded.

Successive 12-month time periods were established (year 1: from 01-09-2007 to 31-08-2008; year 2: from 01-09-2008 to 31-08-2009; year 3: from 01-09-2009 to 31-08-2010; year 4: from 01-09-2010 to 31-08-2011). During each time period, a patient was considered as presenting type 2 diabetes mellitus if the illness onset date was prior to the established cut-off point (i.e. prior to 01-09-2007, or prior to 01-09-2008 and so on), and in addition, the patient had to have public insurance at the start of the year (although not necessarily for the whole year). Thus, the number of patients considered as having type 2 diabetes mellitus was 116,295 in year 1; 123,991 in year 2; 130,554 in year 3 and 134,421 in year 4. The total study population (people with type 2 diabetes mellitus in any of these years) was 149,015.

### Variables and analysis

The study variables were age, sex and existence of complications and comorbidities (whether or not related to type 2 diabetes mellitus). Hospital discharge records were used to study the incidence of complications (Minimum Basic Data Set [MBDS]) [22]. Hospital admission because of acute myocardial infarction, major amputation or avoidable hospitalisations (Ambulatory Care Sensitive Conditions [ACSC]) [23] was determined separately for each observation period.

To study the chronic diseases, a list of 52 health problems was developed and specific criteria were defined to consider that particular disease as active during the period from 01/09/2010 to 31/08/2011 by adapting a methodology previously reported by other authors [24]. In most cases, criteria were based on considering that a person has a chronic disease because they have been assigned the corresponding diagnosis (for example, hypertension); for some illnesses other criteria were applied: diagnosis or prescription of specific medications (e.g., for hypothyroidism and Parkinson’s); repeated diagnosis over several years (low back pain); any history of the diagnosis together with prescription of specific drugs in the previous year (asthma and epilepsy); diagnosis the previous year or repeated prescriptions over several months (depression and anxiety); or repeated prescriptions to treat the given health problem (treated dyspepsia). Further information of this methodology can be found in previous publications [25]. From such 52 health problems list, diabetes mellitus was excluded, and such group was split in two parts: 1) Related chronic comorbidities, that , according to the bibliography [8], included 7 pathologies: ischaemic heart disease, renal failure, stroke, heart failure, peripheral neuropathy, foot ulcers and diabetic retinopathy; and 2) unrelated chronic diseases, that corresponds to the remaining 44 health problems of the list.

The link to this database was feasible for 93% of patients with type 2 diabetes mellitus in year 4; therefore, the number of people in which these pathologies was established was limited to 126,894.

To analyse the data, the incidence or prevalence of the aforementioned pathologies were estimated in the different population strata according to age and sex. Logistic regression determined the effect of the independent variables (sex and age groups) on the coexistence of such pathologies. To see whether there are differences between sexes in the distribution of diseases, the Pearson  test was performed. Reported *P* values under 0.05 were considered statistically significant.

Statistical calculations were performed using Stata Data Analysis and Statistical Software, Release 12 (StataCorp, LP, College Station, TX, USA).

## Results

### Evolution of the incidence of diabetes-related complications

It was observed that the incidence of diabetes-related complications has declined over the past four years. The number of major amputations performed (-23.8%) and the number of acute myocardial infarctions (-13.6%) have decreased, among others. All this information is contained in Table 1.

**Table 1:** **Incidence of complications of type 2 diabetes over the study period**

	Incidence (%)				
Type 2 diabetes complications	Year 1	Year 2	Year 3	Year 4	Percentage increase from year 1 to 4
Myocardial infarction	0.47	0.43	0.43	0.40	-13.64
Major amputations	0.10	0.10	0.09	0.08	-23.75
Avoidable hospitalisations (all causes)	5.85	5.66	5.41	5.50	-5.99
Avoidable hospitalisations because of DM	0.62	0.60	0.54	0.51	-18.14

### Diabetes-related chronic pathologies

For year 4 of the study, the chronic complications of patients with type 2 diabetes are shown in Table 2. It was observed that the prevalence of renal failure, heart failure, peripheral neuropathy, and foot ulcers was higher in women than in men, while the prevalence of ischaemic heart disease and stroke was higher in men than women.

**Table 2:** **Prevalence and incidence of diabetes-related complications in year 4 (September 2010- August 2011)**

	Prevalence (%)		
Type 2 diabetes complications	Total (N = 134,421)	Males (N = 72,539)	Females (N = 61,882)
Ischaemic Heart Disease	11.45	14.54	7.83
Renal failure	8.37	8.35	8.40
Stroke	6.96	7.42	6.42
Heart Failure	4.32	3.93	4.79
Peripheral neuropathy	1.33	1.24	1.44
Foot Ulcers	1.93	1.76	2.13
Retinopathy	7.18	7.18	7.18
	**Incidence (%)**		
**Type 2 diabetes complications**	**Total (N = 134,421)**	**Male (N = 72,539)**	**Female (N = 61,882)**
Myocardial infarction	0.40	0.50	0.29
Major amputations	0.08	0.09	0.06
Avoidable hospitalisations	5.50	5.81	5.14
Avoidable hospitalisations because of DM	0.51	0.52	0.50

When logistic regressions were applied, women with type 2 diabetes mellitus presented a lower probability than men of suffering from ischaemic heart disease, renal failure, stroke, heart failure, foot ulcers and retinopathy (approximately 58%, 30%, 35%, 14%, 6% and 1% less respectively), and all the analyses were statistically significant except the last two chronic diseases mentioned (Table 3). In addition, women also presented a 32% lower probability of suffering avoidable hospitalisations (*P* < 0.001), a 50% lower probability of myocardial infarction (*P* < 0.001) and a 45% lower probability for major amputations (*P* = 0.004) than men. The only condition where females presented a higher probability (10.4%) of suffering than males was peripheral neuropathy.

**Table 3:** **Logistic regressions of diabetes-related complications adjusted according to age and sex**

	Ischaemic heart disease		Renal failure		Stroke		Heart failure		Peripheral neuropathy		Foot ulcers		Retinopathy		Avoidable hospitalisations		Myocardial infarction		Major amputations	
Features	OR	***P***value	OR	***P***value	OR	***P***value	OR	***P***value	OR	***P***value	OR	***P***value	OR	***P***value	OR	***P***value	OR	***P***value	OR	***P***value
**Sex**																				
Males (ref)	1.0		1.0		1.0		1.0		1.0		1.0		1.0		1.0		1.0		1.0	
Females	0.4	<0.001	0.7	<0.001	0.7	<0.001	0.9	<0.001	1.1	0.033	0.9	0.113	1.0	0.943	0.7	<0.0001	0.5	<0.001	0.5	0.004
**Age bands**																				
Range 35-39 (ref)	1.0		1.0		1.0		1.0		1.0		1.0		1.0		1.0 (ref)					
Range 40-44	2.5	0.013	1.1	0.779	2.7	0.111	4.1	0.181	1.7	0.317	1.3	0.572	1.0	0.854	1.1	0.681	1.0(ref)			
Range 45-49	4.5	<0.001	1.5	0.181	5.6	0.004	7.8	0.043	2.1	0.115	1.6	0.348	1.7	0.003	0.9	0.73	0.98	0.976		
Range 50-54	8.2	<0.001	1.6	0.085	7.7	<0.001	9.0	0.029	2.6	0.040	2.3	0.077	1.6	0.004	1	0.881	1.4	0.481	1.0 (ref)	
Range 55-59	10.7	<0.001	2.2	0.004	10.3	<0.001	14.2	0.008	2.6	0.040	1.9	0.153	1.9	<0.001	1.1	0.718	1.3	0.61	1.3	0.678
Range 60-64	11.6	<0.001	3.0	<0.001	14.5	<0.001	19.5	0.003	2.8	0.022	2.5	0.045	2.1	<0.001	1.2	0.353	1.2	0.705	0.5	0.365
Range 65-69	13.9	<0.001	4.1	<0.001	18.8	<0.001	23.8	0.002	3.0	0.015	2.6	0.038	2.2	<0.001	1.4	0.114	1.2	0.747	1.6	0.445
Range 70-74	16.8	<0.001	6.1	<0.001	25.5	<0.001	35.8	<0.001	3.0	0.016	3.5	0.006	2.2	<0.001	1.8	0.006	1.5	0.349	3.1	0.069
Range 75-79	21.0	<0.001	10.0	<0.001	33.6	<0.001	62.6	<0.001	3.0	0.015	4.8	0.001	2.3	<0.001	2.8	<0.001	2.2	0.086	2.2	0.217
Range 80-84	23.7	<0.001	14.2	<0.001	48.1	<0.001	94.9	<0.001	3.0	0.015	6.9	<0.001	2.2	<0.001	3.8	<0.001	2.5	0.044	3.8	0.03
Range 85 and over	26.5	<0.001	21.6	<0.001	65.5	<0.001	156.3	<0.001	2.2	0.083	10.5	<0.001	1.5	0.011	5.5	<0.001	3.5	0.007	3.7	0.037

OR = Odds ratio; ref = reference. Statistically significant subgroups are those with *P* value <0.05.

Age was also an important factor in the complications (Table 3). The probability of suffering from one of these complications increased significantly with age. Since in myocardial infarction there are no people in the age group 35-39, the following group was taken as reference; the same applied to major amputations where the 50-54 age group was taken as reference. Compared to the 35-39 age group, the over 85 age group was not statistically significant for peripheral neuropathy. Moreover, for myocardial infarction and major amputations only age over 79 years was statistically significant for the analysis.

### Unrelated chronic conditions

The relationship with multimorbidity in year 4 of the study for this group of patients is shown in Table 4. It shows the percentage of patients in relation to the number of pathologies they suffer, where 1 represents diabetes type 2, 2 represents diabetes plus one chronic disease, and so on. For both sexes it is observed that two chronic diseases plus diabetes is the most common combination. Almost 30% suffer from five or more chronic diseases, and only 9.64% are free from other pathologies.

**Table 4:** **Number (proportion) of people with type 2 diabetes in relation to number of chronic diseases**

Number of pathologies	Total N (%)	Male (N)	Female (N)
1	12,031 (9.64)	7,849 (11.65)	4,182 (7.28)
2	26,946 (21.59)	15,676 (23.27)	11,270 (19.63)
3	27,312 (21.89)	14,683 (21.80)	12,629 (21.99)
4	21,230 (17.01)	10,795 (16.03)	10,435 (18.17)
5	14,664 (11.15)	7,224 (10.73)	7,440 (12.96)
6	9,139 (7.32)	4,487 (6.66)	4,652 (8.10)
7	5,749 (4.61)	2,779 (4.13)	2,970 (5.17)
8	3,446 (2.76)	1,690 (2.51)	1,756 (3.06)
9	1,992 (1.60)	1,021 (1.52)	971 (1.69)
10 or more	2,272 (1.82)	1,152 (1.71)	1,120 (1.95)

Table 5 shows the prevalence of patients with type 2 diabetes, in general and according to sex, who suffer from each of the 44 chronic diseases. Overall, the most common diseases were hypertension (73.71%), dyspepsia (13.81%), anxiety (12.75%), and degenerative joint disease (11.72%). Among men the study revealed hypertension (73.23%), prostatic hypertrophy (13.48%), atrial fibrillation (12.15%) and COPD (12.09%) as the most common diseases. Among women, the most prevalent conditions were hypertension (73.54%), anxiety (12.75%), dyspepsia (16.7%), degenerative joint disease (15.79%) and depression (14.78%). In patients with type 2 diabetes, no sex differences for deafness (*P* = 0.07), immunological disease (*P* = 0.25), chronic haematological disease (*P* = 0.09), epilepsy (*P* = 0.46), chronic sinusitis (*P* = 0.89), learning disability (*P* = 0.12) and ADHD (*P* = 0.31) were found.

**Table 5:** **Prevalence of 44 chronic diseases in type 2 diabetes patients**

Chronic disease	Overall (%)	Males (%)	Females (%)	Differences between sexes
Hypertension	73.71%	73.23%	73.54%	<0.001
Dyspepsia	13.81%	11.36%	16.70%	<0.001
Prostatic hypertrophy	—	13.48%	—	—
Anxiety	12.75%	8.54%	18.10%	<0.001
Degenerative joint disease	11.72%	7.79%	15.79%	0.002
Atrial Fibrillation	10.40%	12.15%	8.54%	<0.001
Depression	9.77%	5.47%	14.78%	<0.001
Malignant neoplasms	8.86%	11.17%	6.96%	<0.001
Chronic heart disease, other	8.57%	10.77%	6.09%	<0.001
Emphysema, chronic bronchitis, COPD	8.23%	12.09%	4.50%	<0.001
Hypothyroidism	6.22%	2.31%	11.31%	<0.001
Low back pain	5.82%	4.34%	7.65%	<0.001
Dementia	5.05%	4.17%	5.80%	<0.001
Osteoporosis	4.46%	0.83%	8.26%	<0.001
Gout	3.72%	5.75%	1.44%	<0.001
Deafness	3.50%	3.52%	3.41%	0.071
Asthma (currently under treatment)	3.26%	1.99%	4.78%	<0.001
Chronic hepatopancreatic diseases	3.25%	3.91%	2.29%	<0.001
Diverticular disease	2.80%	2.58%	3.03%	<0.001
Peripheral vascular disease	2.50%	3.96%	0.97%	<0.001
Rheumatoid arthritis and others	2.40%	2.10%	2.78%	<0.001
Errors of metabolism and chromosomopathies	2.15%	2.51%	1.69%	<0.001
Parkinson’s disease	1.88%	1.83%	1.99%	<0.001
Muscular dystrophy or paralysis	1.53%	1.73%	1.33%	0.005
Schizophrenia	1.50%	1.24%	1.81%	<0.001
Chronic constipation	1.07%	1.04%	1.17%	<0.001
Alcohol abuse	1.00%	1.46%	0.40%	<0.001
Viral Hepatitis	0.81%	0.84%	0.71%	<0.001
Psoriasis or eczema	0.76%	0.87%	0.63%	<0.001
Immunological diseases	0.63%	0.66%	0.63%	0.245
Irritable bowel syndrome	0.60%	0.38%	0.87%	<0.001
Bronchiectasis	0.59%	0.75%	0.47%	0.003
Chronic Haematological Diseases	0.58%	0.60%	0.59%	0.085
Epilepsy (currently under treatment)	0.55%	0.58%	0.53%	0.457
Inflammatory bowel disease	0.50%	0.52%	0.47%	0.040
Post-transplant state	0.37%	0.44%	0.26%	<0.001
Chronic Sinusitis	0.26%	0.25%	0.28%	0.891
Learning Disabilities	0.13%	0.11%	0.17%	0.115
HIV	0.12%	0.15%	0.06%	<0.001
Migraine	0.11%	0.04%	0.23%	<0.001
Substance Abuse	0.11%	0.14%	0.05%	<0.001
Multiple sclerosis	0.07%	0.05%	0.10%	0.033
Anorexia, bulimia	0.03%	0.01%	0.06%	<0.001
ADHD	0.005%	0.002%	0.01%	0.311

Stratified by sex and all prevalence is adjusted by age and sex (in overall case). Statistically significant differences are those with *P* value <0.05.

ADHD: Attention Deficit Hyperactivity Disorder; COPD: Chronic Obstructive Pulmonary Disease; HIV: Human Immunodeficiency Virus.

## Discussion

Compared with other studies [26–28], we have obtained lower rates of incidence for avoidable hospitalisations, acute myocardial infarction and major amputations. Moreover, as Vamos *et al.* [26], we have found that sex was statistically significant for major amputation and women presented a 45% lower probability than men of being affected; the same applied to myocardial infarction (50% lower) and for avoidable hospitalisations (32% lower).

In this study, we could see a reduction in the rate of incidence of diabetes-related complications from 2007 to 2011. Adequate control of type 2 diabetes risk factors is high in the Basque population [29]. In addition, other studies have reported decreasing incidence of several complications such as amputations [26, 30, 31] and preventable hospitalisations [32]. Vamos *et al.* [26] found that the incidence for amputations decreased by 9.1% in the four-year study period and a series of studies.

The outcomes of chronic complications, such as stroke and ischaemic heart disease are similar to those observed in other Spanish studies [33, 34]. Men had a higher prevalence than women of ischaemic heart disease and stroke. Indeed, sex was statistically significant for all analyses except foot ulcers and retinopathy; women reported approximately a 58%, 30%, 35% and 14% lower probability than men of developing ischaemic heart disease, renal failure, stroke and heart failure, respectively. Furthermore, the probability of suffering one of these complications increased significantly with age.

The prevalence of two or more chronic diseases in addition to type 2 diabetes mellitus was 68.8%. This was equivalent to that recorded in a cohort of Irish primary care patients (68.4%) [17]. In our analysis of sex-related differences for multimorbidity, men presented a greater prevalence of hypertension, prostatic hypertrophy, atrial fibrillation and COPD; women presented greater prevalence of hypertension, anxiety, dyspepsia, degenerative joint disease and depression.

Our study has several strengths. First, Osakidetza is a healthcare system with universal coverage. This makes it possible for our study to include almost all the population with type 2 diabetes in the geographical area under study; selection bias is thus avoided. Second, it makes use of a database which contains information from primary care, specialty care, hospital care, and prescriptions. This is relevant, as other authors have established that the use of just one source can produce inaccurate calculations [35, 36], while the complementary use of various sources contributes to a better description of the persons’ health problems [37].

The study limitations correspond to the use of administrative databases, which only contain information about patients who seek healthcare. Therefore, the prevalence of complications can only reflect known cases and exclude cases not known by the patient or healthcare personnel.

Type 2 Diabetes Mellitus is a disease associated with reduced quality of life due to associated chronic complications that develop throughout the natural development of the disease. Adequate metabolic control may help reduce the risks of complications over the long-term. The epidemiological information available in the BAC gives an idea of its importance and the growing impact on the population and health services.

This study provides relevant epidemiological information about the prevalence and incidence of diabetes-related complications, in addition to, multimorbidity in this patient group. Moreover, it has been utilised to confirm differences between sex and age that could be employed to define interventions focused on certain patient profiles. Moreover, it could even be used as a support for a future diabetes registry to improve the disease monitoring and research in our community.

## Conclusions

The high prevalence of multimorbidity and the diversity of chronic conditions complicate disease management in this group and show the need for a holistic approach in its clinical care and education. Furthermore, the need to educate patients regarding self-care and to pay special attention to the implications of polypharmacy must be emphasised [38]. In the same way, there is a need to perform further research to reach a consensus on developing Clinical Practice Guidelines focused on patients with multiple pathologies, thereby reducing the risk of inappropriate clinical management and patient safety issues such as drug interactions.

Chronic complications of diabetes have a relevant impact on the population with type 2 diabetes. Therefore, an expansion of epidemiological knowledge and improved disease monitoring should be a public health priority, to address the social and health implications and to develop interventions that enable better management of these patients.

## Abbreviations

ACSC: Ambulatory Care Sensitive Condition
ATC: Anatomical, Therapeutic, Chemical classification system
BAC: Basque Autonomous Community
CVRF: Cardiovascular Risk Factor
ICD: International Classification of Disease
MBDS: Minimum Basic Data Set
PREST: Basque Country population stratification programme
SNS: Spanish National Health System.
